# Conversion of stem cells from apical papilla into endothelial cells by small molecules and growth factors

**DOI:** 10.1186/s13287-021-02350-5

**Published:** 2021-05-03

**Authors:** Baicheng Yi, Tian Ding, Shan Jiang, Ting Gong, Hitesh Chopra, Ou Sha, Waruna Lakmal Dissanayaka, Shaohua Ge, Chengfei Zhang

**Affiliations:** 1 Restorative Dental Sciences, Faculty of Dentistry, The University of Hong Kong, Hong Kong Special Administrative Region, China; 2 Shenzhen Institute of Research and Innovation, The University of Hong Kong, Shenzhen, China; 3 Department of Periodontology, School and Hospital of Stomatology, Cheeloo College of Medicine, Shandong University; Shandong Key Laboratory of Oral Tissue Regeneration; Shandong Engineering Laboratory for Dental Materials and Oral Tissue Regeneration, No.44-1 Wenhua Road West, Jinan, Shandong China; 4 School of Dentistry, Shenzhen University Health Science Center, Shenzhen, China; 5 Applied Oral Sciences & Community Dental Care, Faculty of Dentistry, The University of Hong Kong, Hong Kong Special Administrative Region, China

**Keywords:** SCAP, Endothelial differentiation, Small molecules, Chemical reprogramming, Angiogenesis

## Abstract

**Objectives:**

Recently, a new strategy has been developed to directly reprogram one cell type towards another targeted cell type using small molecule compounds. Human fibroblasts have been chemically reprogrammed into neuronal cells, Schwann cells and cardiomyocyte-like cells by different small molecule combinations. This study aimed to explore whether stem cells from apical papilla (SCAP) could be reprogrammed into endothelial cells (ECs) using the same strategy.

**Materials and methods:**

The expression level of endothelial-specific genes and proteins after chemical induction of SCAP was assessed by RT-PCR, western blotting, flow cytometry and immunofluorescence. The in vitro functions of SCAP-derived chemical-induced endothelial cells (SCAP-ECs) were evaluated by tube-like structure formation assay, acetylated low-density lipoprotein (ac-LDL) uptake and NO secretion detection. The proliferation and the migration ability of SCAP-ECs were evaluated by CCK-8 and Transwell assay. LPS stimulation was used to mimic the inflammatory environment in demonstrating the ability of SCAP-ECs to express adhesion molecules. The in vivo Matrigel plug angiogenesis assay was performed to assess the function of SCAP-ECs in generating vascular structures using the immune-deficient mouse model.

**Results:**

SCAP-ECs expressed upregulated endothelial-specific genes and proteins; displayed endothelial transcriptional networks; exhibited the ability to form functional tubular-like structures, uptake ac-LDL and secrete NO in vitro; and contributed to generate blood vessels in vivo. The SCAP-ECs could also express adhesion molecules in the pro-inflammatory environment and have a similar migration and proliferation ability as HUVECs.

**Conclusions:**

Our study demonstrates that the set of small molecules and growth factors could significantly promote endothelial transdifferentiation of SCAP, which provides a promising candidate cell source for vascular engineering and treatment of ischemic diseases.

**Supplementary Information:**

The online version contains supplementary material available at 10.1186/s13287-021-02350-5.

## Introduction

Postnatal angiogenesis plays an essential role in the recovery of ischemic tissues and survival of engineered tissue constructs after implantation [1, 2]. During this process, locally elevated angiogenic factors, such as vascular endothelial growth factor (VEGF) and basic fibroblast growth factor (b-FGF), activate endothelial cells’ proliferation, migration and self-assembling to form primitive vascular networks, which is followed by stepwise mural cell recruitment and stabilization of the nascent vessels [3]. In the developed vasculature, endothelial cells (ECs) not only are the principal components of forming the inner layer of blood vessels, but also associated with smooth muscle cells and pericytes for maintaining the physiological functions of blood vessels [4]. Therefore, the role of ECs in vascular development and vascular tissue engineering would never be overemphasized.

For vascular tissue engineering and its translational application, the first step is to obtain adequate number of ECs. Autologous endothelial cells like human umbilical vein endothelial cells (HUVECs) and human microvascular endothelial cells (HMECs) are the most suitable cell sources for vascularization. But their scarce availability and low proliferation rates limit the large-scale application of these cells [5]. Endothelial progenitor cells (EPCs) derived from the human peripheral blood, bone marrow, and umbilical cord blood have been investigated for augmenting angiogenesis [6]. However, limited yield of EPCs, such as 0.01% from the peripheral blood, 0.05% from the bone marrow and 0.2 to 1% in the umbilical cord blood [6, 7], restricts the potential in translational applications. There are also some concerns on the effects of long-term cryopreservation on EPCs’ functions and in vivo efficacies [8]. Generation of ECs from pluripotent stem cells (PSCs), such as embryonic stem cells (ESCs) and induced pluripotent stem cells (iPSCs), has been reported [9, 10]. However, ethical issues, tumorigenicity and the concerns of genome instability hinder the clinical application of these cells [11–13]. Alternatively, mesenchymal stem cells (MSCs) extracted from the bone marrow, adipose tissues and dental tissues are relatively easy to obtain in sufficient quantities for translational application. Particularly, dental-derived stem cells, which express several pluripotency markers not usually expressed by other adult stem cells, such as SOX2, NANOG and OCT4, could be a potential substitute cell source for endothelial differentiation [14].

Direct reprogramming, defined as the direct generation of targeted cell types from another cell type without passing through an intermediate pluripotent stage, is commonly achieved by overexpression of cell/tissue-specific transcription factors [15]. For example, human fibroblasts have been successfully reprogrammed into ECs by transfection of FOXO1, ETV2, KIF2, TAL1 and LMO2 or even single factor ETV2 [16, 17]. The reprogramming process could be achieved via a chemical cocktail composed of epigenetic modulators, signalling pathway regulators and other factors that induce the characteristics of the designated cell types [18, 19]. Neuronal cells, Schwann cells and cardiomyocyte-like cells have been successfully reprogrammed from human fibroblasts by different small molecule compound combinations [20–22]. SCAP, a unique type of mesenchymal stem cells derived from the tooth apical papilla, have been shown to possess higher multipotent differentiation potential and constitutively expressing pluripotency markers [14, 23]. Therefore, it is reasonable to hypothesize that SCAP could differentiate into endothelial-like cells via the chemical approach.

In this study, we identified a chemical protocol composed of five small molecule compounds (VPA, CHIR99021, Repsox, Forskolin and Y-27632) and three key growth factors (VEGF, BMP-4 and 8-Br-cAMP), which could efficiently induce SCAP into EC-like cells that possess the vascular identity in vitro and in vivo. This approach could be used as a prospective basic strategy for endothelial induction, through which ECs can be produced for vascular engineering and management of ischemic diseases.

## Materials and methods

### Cell culture

SCAP were kindly provided as a gift by Dr. Anibal Diogenes (Department of Endodontics, University of Texas Health Science Center). The cells were cultured and expanded in α-MEM supplemented with 10% FBS, 100 g/L streptomycin and 100000 U/L penicillin (Gibco, Carlsbad, CA, USA). When reaching 80% confluence, the cells were trypsinized using 0.25% trypsin (Gibco), harvested and serially passaged. The cells from 3rd to 6th passage were utilized for experiments. SCAP were characterized by flow cytometric analysis of the expressions of CD45, CD73, CD90 and CD105 as well as multi-lineage differentiation assays (Additional file 1: Appendix Figure 1).

HUVECs and pericytes were commercially purchased (ScienCell Research Laboratories, San Diego, CA) and cultured in endothelial cell medium (ECM) and pericyte medium (PM) respectively (ScienCell Research Laboratories) at 37°C with 5% CO_2_. Cells below passage 6 were used for further experiments.

### Generation of endothelial-like cells from SCAP with small molecules

SCAP (3×10^5^) were seeded on 0.1% gelatin-coated 6-well plates in α-MEM and incubated at 37°C in a humidified atmosphere with 5% CO_2_. At 40–50% cell confluence, the culture medium was changed to EGM-2 (Lonza, Walkersville, MD, USA), plus 50 ng/mL VEGF (rhVEGF165, Peprotech, NJ, USA), 20 ng/mL BMP-4 (R&D Systems, Minneapolis, MN, USA) and the chemical cocktail 0.5 mM VPA (Sigma-Aldrich, St. Louis, MO, USA), 3 μM CHIR99021 (Stemgent, Beltsville, MD, USA), 1 μM Repsox (Selleck, Houston, TX, USA), 10 μM Forskolin (Caymen, Ann Arbor, MI, USA), 5 μM Y-27632 (Sigma-Aldrich) for a 4-day induction, and then BMP-4 was replaced by 100 μM 8-Br-3,5-cAMP (Sigma-Aldrich) in the next 4 days (Appendix Table 1). The medium was changed every 2 days. The obtained cells are passaged and cultured in a maintenance medium composed of EGM-2 and 0.5 mM VPA, 3 μM CHIR99021, 1 μM Repsox, 10 μM Forskolin and 5 μM Y-27632.

### Quantitative real-time polymerase chain reaction, western blotting, flow cytometry immunofluorescence and RNA sequencing

See Additional file 1 for detailed materials and methods. The primer sequences and antibodies used in this study are listed in Additional file 1: Appendix Tables 2 and 3.

### Tube-like structure formation assay

To assess the angiogenic capacity of SCAP-ECs, a tubular formation assay was conducted on Matrigel. Briefly, 48-well cell culture plates were coated with 150-μL chilled Matrigel solutions (Corning, NY, USA) per well using a precooled pipette. The 48-well plates were then incubated in a cell culture incubator for 30 min to allow the solidification of the Matrigel. SCAP only, SCAP in EGM2, SCAP-ECs and HUVECs were collected, counted and resuspended in EGM-2. Subsequently, 3× 10^4^ cells of a single-cell suspension were reseeded onto the top of the solidified Matrigel within each well. Images were captured at different time points (12h, 24h) with a digital camera attached to an inverted microscope (Olympus, Tokyo, Japan). The number of nodes, meshes, junctions and total tube length were quantified using ImageJ analyser. Pericytes provide support around ECs and modulate the EC behaviours including the formation of endothelial cell-cell junctions. In order to evaluate the integration effect, SCAP-ECs and pericytes were labelled using CellTracker® fluorescent probes (Life Technologies, CA, USA) and seeded on the Matrigel surface at a density of 3 × 10^4^ cells/well and 7.5× 10^3^ cells/well respectively. Images were captured at 12h.

### Acetylated low-density lipoprotein uptake

To assess acetylated low-density lipoprotein (ac-LDL) uptake, SCAP-ECs were incubated with Dil ac-LDL (Invitrogen, Waltham, MA) at 37°C for 6 h at a concentration of 10 μg/mL then fixed with 4% paraformaldehyde and counterstained with DAPI. To detect ac-LDL uptake, cells were imaged using a fluorescence microscope (Olympus).

### Nitric oxide secretion detection

The ability of the cells to produce nitric oxide (NO) was assessed by measuring the concentration of NO in the culture medium containing 2 ng/mL VEGF (rhVEGF165, Peprotech) and 1μM Calcium Ionophore A23187 (Sigma-Aldrich) with the use of the NO detection kit (Invitrogen, Waltham, MA) according to the manufacturer’s instructions. The amount of nitrate was determined by converting it to nitrite, followed by the colorimetric determination of the total concentration of nitrite as a coloured azo dye product of the Griess reaction that absorbed visible light at 540 nm with the use of a microplate reader (MTX Lab Systems, WA).

### Expression of adhesion molecules in an inflammatory environment

To detect the expression of adhesion molecules in a pro-inflammatory environment, SCAP-ECs were incubated with ECM containing 1μg/mL *Escherichia coli* LPS (Sigma-Aldrich) for 24 h. The expression of interleukin-6 (IL-6) and interleukin-8 (IL-8) mRNA in SCAP-ECs and HUVECs after stimulation were detected by RT-PCR. Western blot was utilized to determine the expression of intercellular adhesion molecule 1 (ICAM-1) and vascular cell adhesion molecule 1 (VCAM-1) of SCAP-ECs, with HUVECs as the positive control.

### Transwell migration assay

Twenty-four-well Transwell Permeable Supports with 8-μm pores (Corning, NY, US) were used. SCAP-ECs and HUVECs were trypsinized, resuspended by ECM with 0.1% FBS and seeded on the upper chamber of inserts at a density of 8 × 10^4^/200 μL. Then, 600μL of ECM with 5% FBS was added to the lower compartment of each well. After 24 h, the medium was removed and the upper side of the permeable membrane was cleansed with a cotton swab to remove the cells that have not passed through the membrane. The cells on the lower surface of the membrane were fixed by 4% paraformaldehyde for 30 min and stained with 0.1% crystal violet (Sigma) for 20 min. The number of cells on the lower surface of the permeable membrane was calculated under the inverted microscope (Olympus) and an average of 5 fields per each well was taken as the final value.

### CCK-8 assay

To evaluate the proliferative ability of SCAP-ECs, the cell counting kit-8 (CCK-8, Sigma-Aldrich) assay was performed. Cell suspensions (5 × 10^3^ cells/well) of SCAP-ECs or HUVECs were added into 96-well plates at a volume of 100 μL per well. The SCAP-ECs were cultured in a maintenance medium and HUVECs were cultured in ECM with 5% FBS. At indicated time points (day 1, 3, 5 and 7), the culture medium was replaced with a medium containing CCK-8 solution and incubated at 37°C in 5% CO_2_ for 2 h. The absorbance readings of the wells were then measured on a microplate reader (MTX Lab Systems) at 450 nm to assess the number of viable cells in each well. The readings of the background absorbance of the wells containing medium and CCK-8 solution without cells were also measured. Cell proliferation was represented as mean ± SD of absorbance readings of 6 wells from each group.

### In vivo Matrigel angiogenesis assay

All animal experiment protocols were approved by the Committee on the use of live animals in the Stomatology School of Shandong University, Shandong, China. Briefly, a total of 4 × 10^6^ cells (group 1, SCAP; group 2, SCAP in EGM-2 and with + SCAP 1:1; group 3, SCAP-ECs + SCAP 1:1; group 4, HUVEC + SCAP 1:1) were resuspended in 400 μL Matrigel (Corning, NY, USA). Three mice were allocated to each group and two Matrigel plugs were bilaterally injected into each mouse. A pre-chilled syringe with a 25-gauge needle was used to inject the Matrigel/cell mixture into the subcutaneous space of 6-week-old NOD/SCID mice. After 7 days, the implants were retrieved, fixed in 4% formaldehyde for 24h, embedded in paraffin and sectioned (5-μm thickness). Haematoxylin and eosin (H&E) staining was performed to detect the presence of newly formed blood vessels in the Matrigel. For visualization of luminal structures derived from SCAP-ECs, the sections were stained with immunofluorescence for a human-specific vWF antibody (Sigma-Aldrich) and then incubated with Alexa Fluor-594-conjugated secondary antibody (Invitrogen, Carlsbad, CA). A mouse-specific CD31 antibody (Abcam) was used to demonstrate the interaction of human SCAP-derived ECs and host cells. The images were captured with an Olympus IX-81 confocal laser scanning microscope (Olympus, Tokyo, Japan).

### Statistical analysis

All experiments were performed in triplicates, and the results were presented as mean ± standard deviation (SD). Statistical comparisons were performed by Student’s *t* test and 1-way analysis of variance (ANOVA), with *P* values less than 0.05 considered statistically significant.

## Results

### Reprogramming SCAP into endothelial-like cells by small molecule cocktail

**Fig. 1:**
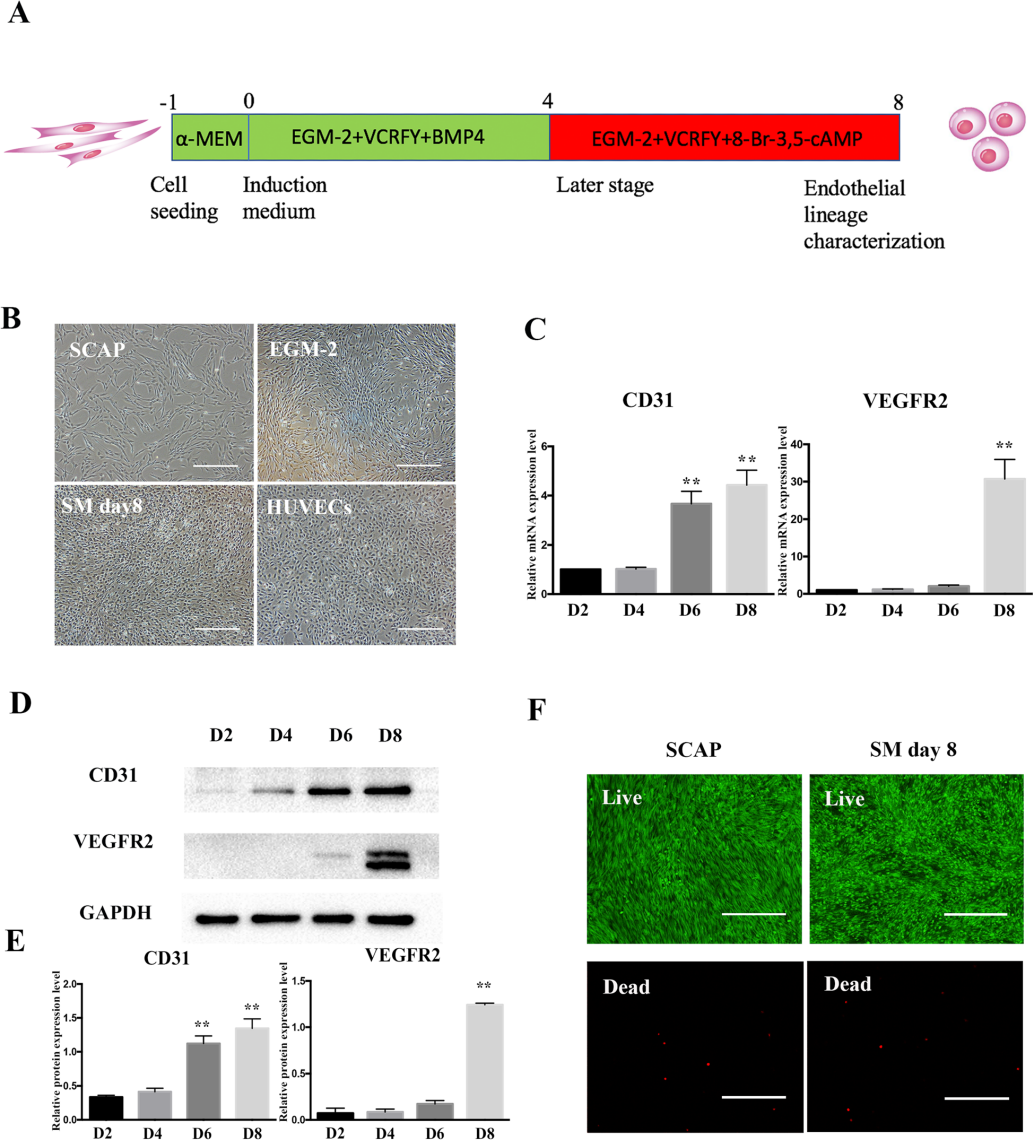
The induction of SCAP into endothelial-like cells using small molecules and growth factors. **a** Schematic diagram of the induction protocol. **b** Representative morphological changes of SCAP after SM exposure. Scale bar = 500 μm. **c** The gene expression levels of CD31 and VEGFR2 in SCAP during SM treatment. All of the results represent the mean ± SEM of 3 independent experiments (*n* = 3). **d**, **e** The protein expression levels of CD31 and VEGFR2 in SCAP during SM treatment. The band intensities were analysed using ImageJ software; GAPDH was used as an internal control. All of the results represent the mean ± SEM of 3 independent experiments (*n* = 3). **f** Assessment of the viability of SCAP after SM exposure using live/dead assay. Scale bar = 500 μm. ^*^P < .05, ^**^P < .01

We have recently demonstrated that TGF-β signalling inhibitor SB431452 could significantly enhance the capacity of VEGF to induce SHED differentiation into ECs [24]. This study indicated that small molecule compounds modulating key cell signalling pathways could drive dental stem cells towards endothelial differentiation. However, the conversion of SCAP into endothelial lineage by using a cocktail of small molecules has never been investigated. We first used VPA, CHIR99021, Repsox, Forskolin and Y-27632 (VCRFY) with EGM-2 supplemented with 50 ng/mL VEGF as a basic chemical endothelial-induction system. After inducing for 8 days, SCAP changed from spindle to round shape and overexpressed CD31 protein. However, the essential endothelial marker VEGFR2 could not be detected (Additional file 1: Appendix Figure 2A and B). BMP-4 is a key cytokine in the commitment of mesodermal progenitors for pluripotent stem cells (PSCs) and is often used for the early stage of EC differentiation [25]. 8-Br-cAMP could enhance endothelial reprogramming efficiency in the later stage [17, 26]. Therefore, in order to improve the reprogramming efficiency, we sequentially introduced BMP-4 and 8-Br-cAMP, which led to VEGFR2 protein expression in the induced SCAP. Accordingly, the final induction scheme was optimized as follows: EGM-2 plus VEGF, BMP4 and the chemical cocktail VCRFY for a 4-day induction, and BMP4 was replaced by 8-Br-cAMP in the next 4 days (Fig. 1a). At the end of the chemical induction, compared to SCAP, the morphology of the obtained cells gained a significant change, which is a rounded shape that resembles primary endothelial cells (Fig. 1b). After 8 days of induction, small molecules upregulated the mRNA and protein expression levels of CD31 and VEGFR2 in a time-dependent manner (Fig. 1c–e). Collectively, the proposed compounds, including epigenetic modulators, signalling pathway regulators and specific culture medium containing angiogenic growth factors, could induce endothelial differentiation of SCAP. The live/dead assay showed that small molecule cocktail did not significantly affect the cell viability, as compared with the non-treated SCAP (Fig. 1f).

### Characterization of endothelial identity after small molecule cocktail-based lineage conversion of SCAP

**Fig. 2:**
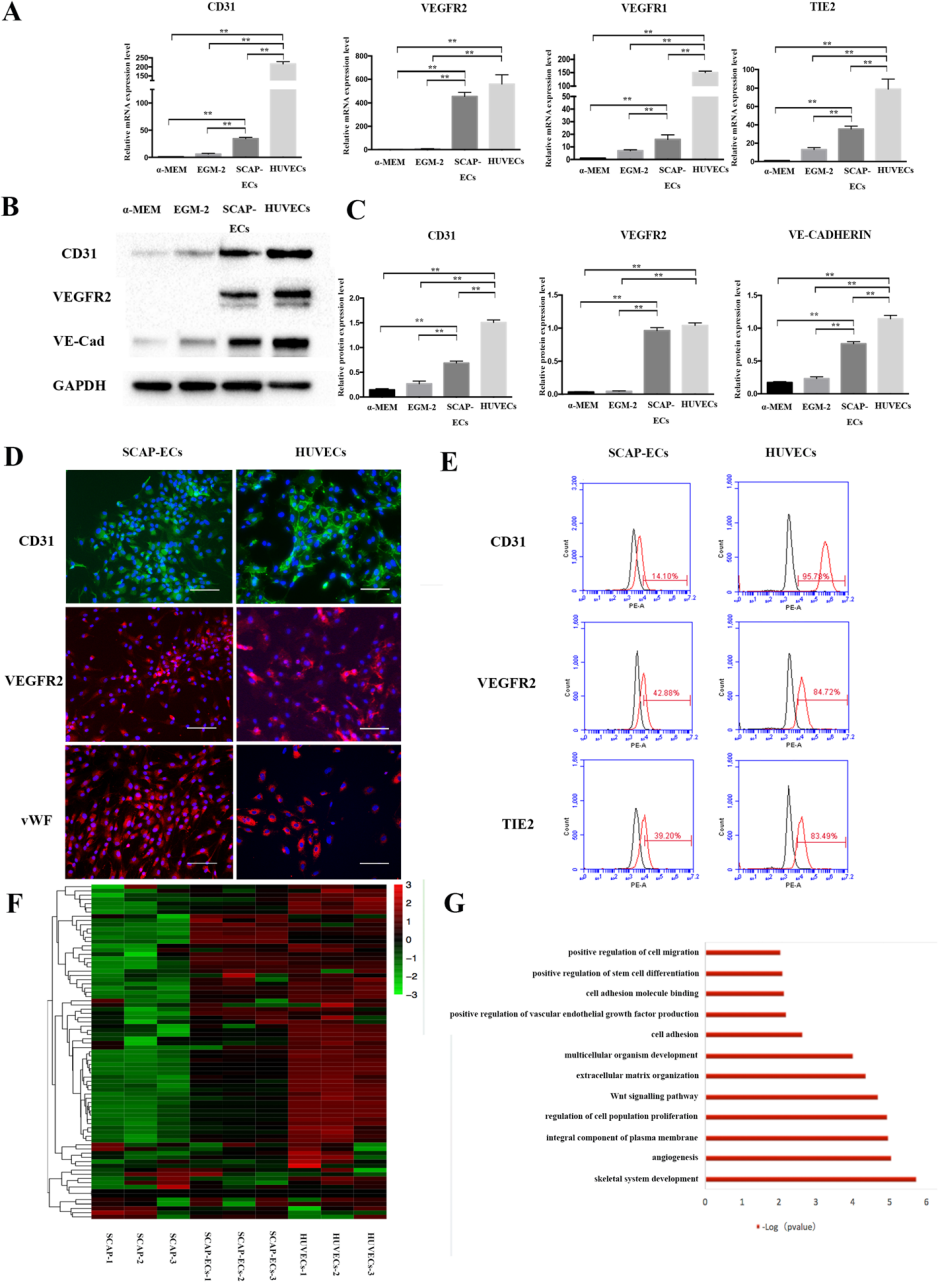
Characterization of endothelial identity after SM-based lineage conversion of SCAP. **a** mRNA expression of endothelial-specific markers in non-treated SCAP, SCAP in EGM2, SCAP-ECs and HUVECs. **b**, **c** Protein expression levels of endothelial-specific markers in SCAP, SCAP in EGM2, SCAP-ECs and HUVECs. **d** The SCAP-ECs were immune-positive for CD31, VEGFR2 and vWF, with HUVECs as control. Scale bar = 100 μm. **e** Flow cytometry analysis of SCAP-ECs showed relatively higher expression levels of VEGFR2, TIE2 and CD31, with HUVECs as control. **f** EC-specific transcriptome profiles of SCAP, SCAP-ECs and HUVECs. **g** GO analysis of upregulated genes of SCAP-ECs versus SCAP. All of the results represent the mean ± SEM of 3 independent experiments (*n* = 3). ^*^P < .05, ^**^P < .01

We first characterized the endothelial identity after chemical reprogramming of SCAP by assessing endothelial-specific gene and protein expressions. Small molecules could significantly upregulate the expression of EC-specific genes (Fig. 2a). After 8 days of induction, SCAP-ECs showed much higher expression of CD31, VEGFR2, VEGFR1 and TIE2, compared with that of SCAP cultured in EGM-2 alone and the non-treatment group. In particular, compared with the non-treatment group, the VEGFR2 mRNA expression of the small molecule induction group was 400 times greater, indicating its strong effect on endothelial lineage induction. Withdrawal of each small molecule would attenuate the expression of VEGFR2 (Additional file 1: Appendix Figure 2C). Small molecule cocktail also significantly enhanced the expression levels of EC-specific proteins CD31, VEGFR2 and VE-CADHERIN compared with the non-treatment group and EGM-2+VEGF group (Fig. 2b, c). Surprisingly, VEGFR2 protein could only be detected in SCAP-ECs. After re-plating on the glass slices, the SCAP-ECs were mainly positive for CD31, VEGFR2 and von Willebrand factor (VWF) (Fig. 2d). Consistent with the western blotting results, the small molecule cocktail could also significantly enhance the protein expression level of EC-specific proteins CD31, VEGFR2 and vWF compared with the non-treatment and EGM-2+VEGF groups (Additional file 1: Appendix Figure 3A). According to flow cytometry analysis, the SCAP-ECs expressed moderately high levels of VEGFR2 (42.88%), TIE2 (39.20%) and CD31 (14.10%) (Fig. 2e), while the cells that were not treated with small molecule compounds showed much lower levels of expression (Additional file 1: Appendix Figure 3B). We further compared transcriptome profiles of SCAP, SCAP-ECs and HUVECs. Heat map analyses demonstrated SCAP-derived ECs showed enriched EC-related gene expression and that the pattern of EC gene expression was closer to HUVECs than to SCAP (Fig. 2f). Gene ontology term enrichment analysis showed that the differentiated genes expressed in SCAP and SCAP-ECs were related to the production of endothelial growth factors, angiogenesis and extracellular matrix organization (Fig. 2g).

### In vitro functional evaluation of the induced endothelial cells

**Fig. 3:**
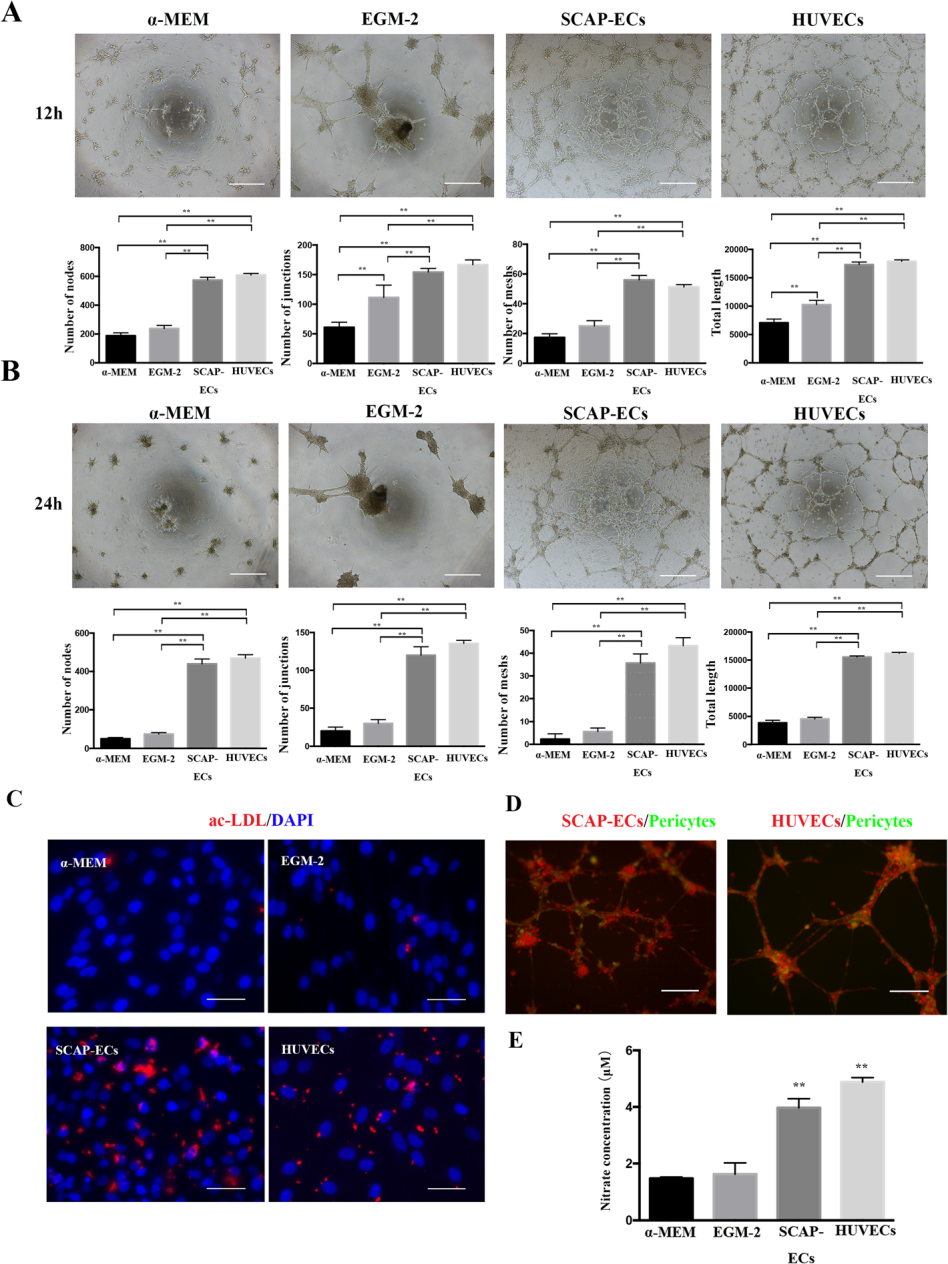
The in vitro functional assessment of SCAP-ECs. **a**, **b** The tube-like structure formation on Matrigel assay. Scale bar = 500 μm. **a** At 12 h, SCAP-ECs showed a significantly higher number of nodes, junctions, meshes and total tube length than the EGM-2 group and SCAP. **b** At 24 h, only SCAP-ECs and HUVECs maintained an intact tubular network. **c** Immunofluorescence for Dil ac-LDL. Ac-LDL uptake was detected in SCAP-ECs. Scale bar = 50 μm. **d** Pericytes (green) were localized along tube-like structures formed by both SCAP-ECs (red) and HUVECs (red). Scale bar = 200 μm. **e** The ability of NO generation in SCAP-ECs, SCAP in EGM-2 group and non-treated SCAP and HUVECs. All of the results represent the mean ± standard deviation of 3 independent experiments (*n* = 3). ^*^P < .05, ^**^P < .01

The ability of induced endothelial cells to generate vascular tube-like networks was assessed by plating undifferentiated SCAP, SCAP cultured in EGM-2 containing 50 ng/mL VEGF, SCAP-ECs and HUVECs on Matrigel. The SCAP-ECs demonstrated elevated vascular tube formation as shown by significantly higher network formation parameters compared with the EGM-2 group and SCAP only. A significantly higher number of nodes, junctions, meshes and total tube length were observed in SCAP-ECs at 12h (Fig. 3a). At 24h, only SCAP-ECs and HUVECs still maintained the tubular network, while the capillary-like structures of the non-treated SCAP or EGM-2 group collapsed (Fig. 3b). Pericytes (green) in both SCAP-ECs (red) group and HUVECs (red) group were localized along tube-like structures, mimicking the architecture of capillary in vivo, where pericytes were wrapped around endothelial cells (Fig. 3d). In addition, SCAP-ECs showed higher levels of ac-LDL uptake compared to the EGM-2 group and SCAPs only (Fig. 3c), with HUVECs as a standard control. In another functional assay to characterize ECs, where the release of nitric oxide was assessed, the SCAP-ECs possessed the ability of generating NO and showed higher NO generation capacity than that of SCAP (Fig. 3e).

### Adhesive molecule expression, migration, maintenance and proliferation of the induced endothelial cells

**Fig. 4:**
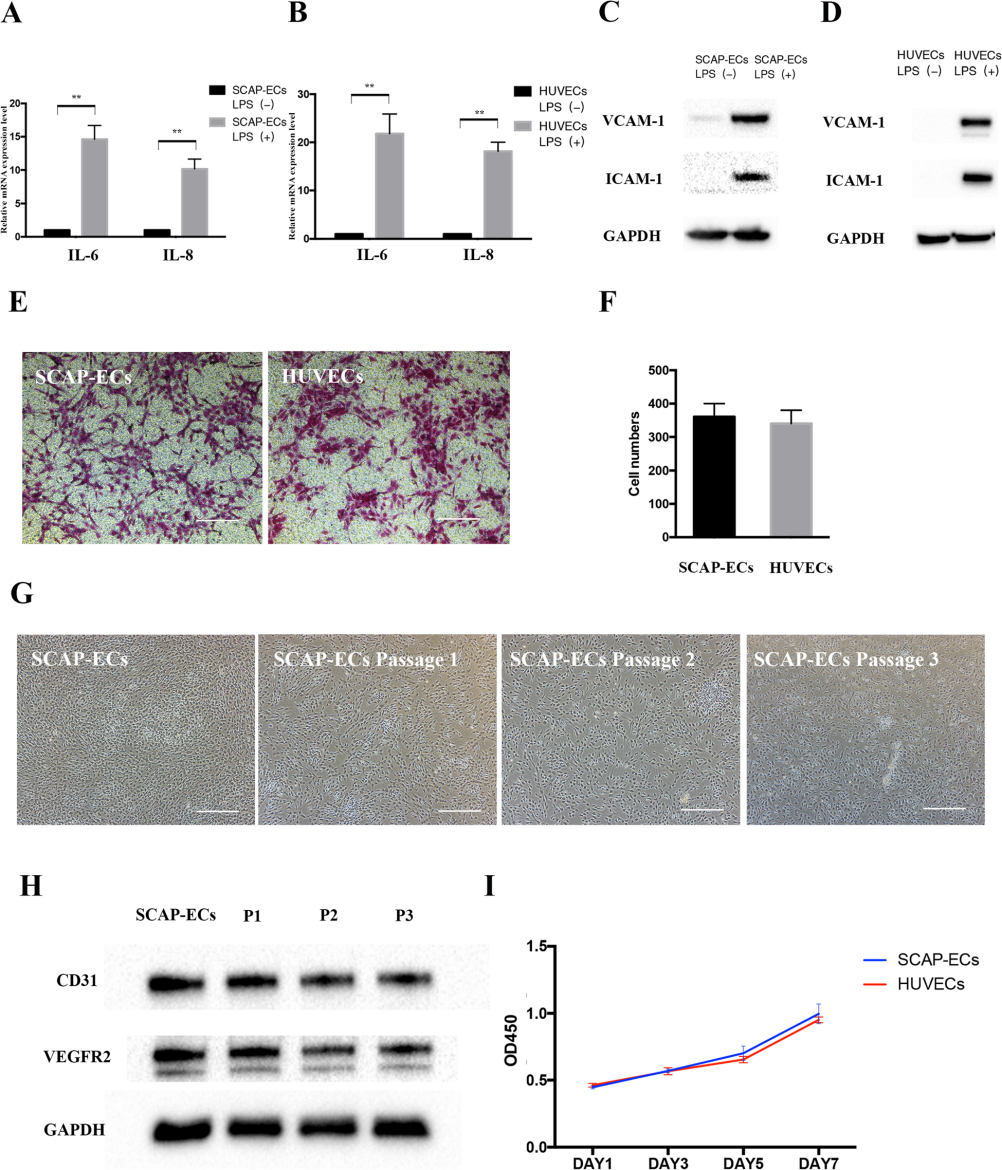
Adhesive molecule expression, migration, maintenance and proliferation of SCAP-ECs. **a**, **b** The gene expression levels of IL-6 and IL-8 in SCAP-ECs and HUVECs after LPS treatment. All of the results represent the mean ± SEM of 3 independent experiments (*n* = 3). **d**, **e** The protein expression levels of VCAM-1 and ICAM-1 in SCAP-ECs and HUVECs after LPS treatment. GAPDH was used as an internal control. **e**, **f** Transwell migration assay did not demonstrate statistical significance of SCAP-ECs and HUVECs in the number of cells migrated through the membrane. Scale bar = 200 μm. Values are presented as mean ± SD. **g** Representative bright field photos of maintenance and passaging of SCAP-ECs. Scale bar = 500 μm. **h** The passage 1 to 3 cells could still express the key endothelial cell markers CD31 and VEGFR2, as demonstrated by western blot. **i** CCK-8 assay did not show statistical significance in OD450 values in SCAP-ECs after digestion and HUVECs. *P < .05, **P < .01

To determine whether SCAP-ECs respond to pro-inflammatory cytokines by adopting a pro-adhesive phenotype, 1 μg/mL LPS was used to mimic the inflammatory environment. After 24 h of LPS stimulation, SCAP-ECs and HUVECs both showed increased IL-6 and IL-8 mRNA levels (Fig. 4a, b). Similar to HUVECs, SCAP-ECs could also express VCAM-1 and ICAM-1protein, as shown by western blot results (Fig. 4c, d). The Transwell migration assay demonstrated no significant difference in the cell migration after 24h between SCAP-ECs group and HUVECs group, implicating that SCAP-ECs showed similar migration ability as HUVECs (Fig. 4e, f). We used a “maintenance medium” composed of EGM-2, VPA CHIR99021, Repsox, Forskolin and Y-27632 (VCRFY) for expanding and culturing the SCAP-ECs. The representative images for passage 1 to passage 3 cells were presented in Fig. 4g, which demonstrated an endothelial cell-like morphology with a rounded shape. The passage 1–3 cells could still express the key endothelial cell markers CD31 and VEGFR2, as demonstrated by western blot (Fig. 4h). SCAP-ECs cultured in the maintenance medium were assessed by the CCK-8 assay at different time points (1, 3, 5 and 7 days), with HUVECs as the positive control. There is no significant difference in the OD450 value between SCAP-ECs and HUVECs at days 1, 3, 5 and 7 (Fig. 4i). After the digestion by trypsin and re-plating on a 96-well plate, SCAP-ECs still showed a similar proliferative ability to HUVECs, implicating their potential in the cell therapy.

### In vivo functional evaluation of the induced endothelial cells

**Fig. 5:**
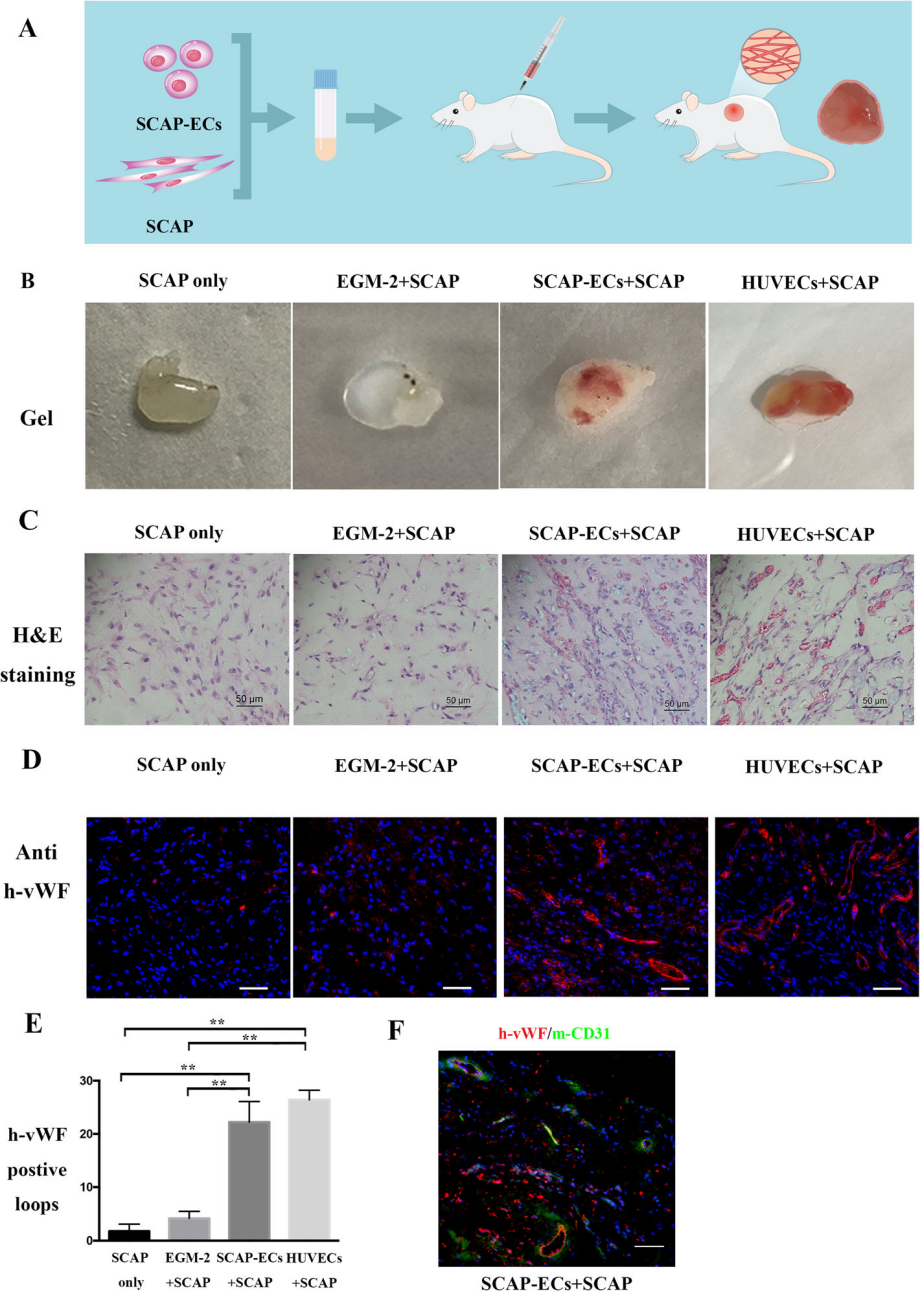
In vivo Matrigel plug assay. **a** A schematic diagram of the experimental flow of the in vivo Matrigel plug assay. **b** Representative macroscopic images of explanted Matrigel plugs (7 days after implantation). **c** H&E-stained sections of Matrigel plugs. **d** Immunofluorescence-stained sections of Matrigel plugs for anti-human vWF (red). Nuclei were counterstained with DAPI (blue). **f** Mouse CD31 (green) and human vWF (red) double staining showed the interaction of human SCAP-derived ECs and host cells in the newly generated blood vessels. All images are representative from 3 animals each group and 2 implants per mouse. Scale bar = 50 μm. ^*^P < .05, ^**^P < .01

To sufficiently assess the function of SCAP-ECs in generating blood vessels, the in vivo Matrigel plug angiogenesis assay was performed. The experimental work flow of the in vivo Matrigel plug angiogenesis assay is shown in Fig. 5a. The plugs were retrieved and analysed 7 days after implantation. The Matrigel encapsulating SCAP + SCAP-ECs was much more vascularized than the plugs encapsulating SCAP only or SCAP + SCAP in the EGM-2+VEGF group, indicating the angiogenic capacity of SCAP-ECs (Fig. 5b). H&E staining showed more blood vessels in the SCAP-ECs than in SCAP only and SCAP + SCAP in EGM-2+VEGF groups. Furthermore, the majority of blood vessels were perfused with erythrocytes, suggesting that these blood vessels were anastomosed to the host vasculature (Fig. 5c, Additional file 1: Appendix Figure 4A). Immunofluorescence staining for human vWF confirmed that the newly formed blood vessels were mainly generated by SCAP-derived ECs (Fig. 5d, Additional file 1: Appendix Figure 4B). Quantitative analysis showed that vWF-positive blood vessel density in the SCAP-EC group was much higher than that of SCAP only or SCAP + SCAP in EGM-2+VEGF groups (Fig. 5e). Mouse CD31 and human vWF double staining showed the interaction of human SCAP-derived ECs and host cells in the newly generated blood vessels (Fig. 5f).

## Discussion

Non-pluripotent human somatic cell lines have been reprogrammed into several cell types by different small molecule combinations [20–22, 27]. Compared to other methods, small molecules have several unique advantages, such as availability, flexible combination based on the targeted cell types, and easy to control in a time and concentration-dependent manner [19]. To the best of our knowledge, there is only one study that used small molecule activators to generate endothelial cells from human fibroblasts [26]. Sayed et al. used a toll-like receptor 3 (TLR3) agonist Poly I:C, which stimulates innate immune signalling, in the endothelial reprogramming protocol. Poly I:C could activate TLR3 to induce global changes in the gene expression and activity of epigenetic modifiers and facilitate the cells to convert into ECs under the specific culture system. Transplantation of the induced endothelial cells (iECs) in the hind limb ischemia mouse model increased the blood flow and capillary density. This study promoted us to explore whether small molecule compounds cocktail-based reprogramming strategy could reprogram dental-derived stem cells into endothelial cells.

For most of the small molecule combinations, no matter what the targeted cell types are, there is a core cocktail, including epigenetic modulators, key signalling pathway regulators and other factors that induce the characteristics of the designated cell types [18, 19]. Therefore, we hypothesized that the combination of core cocktails with specific culture conditions (endothelial differentiation medium) could induce dental stem cells into the endothelial lineage. VPA is the most widely used epigenetic modulator in different chemical reprogramming protocols for overcoming the epigenetic barrier between different types of cells. TGF-β signalling inhibitor Repsox and GSK signalling inhibitor CHIR99021 are most commonly used for suppressing the characteristics of the starting cells. It was reported that if the starting cells are mouse embryonic fibroblasts (MEFs), these compounds which suppress mesenchymal phenotype and promote mesenchymal-to-epithelial transition (MET) are necessary [28, 29].

The chemicals CRFY used in this study were also reported to be involved in the endothelial differentiation of human pluripotent stem cells or dental stem cells. The synergistic modulation of multiple signalling pathways by CRFY might be critical for the endothelial lineage switch. SB431452, an inhibitor of TGF-β1 receptor, could enhance endothelial differentiation of SHED [24]. CHIR99021 inhibits phosphorylation levels of GSK-3β and upregulates the expression of β-catenin, VEGFR2, VE-Cadherin, CD31 and TIE2 on VEGF-induced endothelial differentiation of DPSCs and SHED [30]. Forskolin is commonly used to upregulate the levels of cyclic adenosine monophosphate (cAMP). It was reported that 8-Br-cAMP could enhance the endothelial reprogramming efficiency by 4-fold (3.85% efficiency) as shown by the number of CD31-positive cells at day 28 [17]. Sayed et al. had also used 8-Br-cAMP in their induction protocol and had obtained a 2% efficiency of conversion rate as demonstrated by the number of CD31-positive cells [26]. Furthermore, Rho-associated protein kinase (ROCK) inhibitor Y-27632 could improve differentiation and expansion of embryonic stem cell-derived ECs [31], which was also included in our small molecule combination.

SCAP are a unique type of dental stem cells at a relatively early stage of development confined within the apical papilla. It was reported that angiogenic factors could promote the endothelial differentiation of SCAP. After exposure to an angiogenic induction medium (M199 medium supplemented endothelial cell growth supplement) for 28 days in normoxic condition, SCAP gained the endothelial phenotype in vitro, with upregulation of CD31, vWF, VEGFR2, angiopoietin-1/2 and Tie-1 expression [32]. Koutsoumparis et al. treated SCAP with erythropoietin (rhEPOa) and found that CD31, CDH5 and VEGFR2 were upregulated and MMP2-pathway was activated [33].

In this study, by using the designated small molecules and endothelial-specific differentiation medium, we have successfully and efficiently reprogrammed SCAP into endothelial cells. These reprogrammed cells displayed biological characteristics of ECs. RT-PCR, western blotting, immunofluorescence and flow cytometry data showed that gene and protein expression of endothelial markers were significantly upregulated after small molecule treatment. Endothelial cells derived from dental stem cells are also always assessed by functional tests. Compared to our former methods driving endothelial differentiation of dental stem cells (TGF-β signalling inhibitor [24] and decellularized extracellular matrix of HUVEC [34] treatment), the SCAP-ECs could maintain the tubular structure for a longer time (up to 24h). These induced ECs also showed LDL uptake ability at a comparable level to, and NO production capacity slightly lower to that of HUVECs. In addition, the RNA-sequencing analysis demonstrated that induced ECs share more similar transcriptome to that of HUVECs than non-induced cells. The SCAP-derived ECs could be expanded and cultured in maintenance medium, expressed adhesion molecules with LPS stimulation and showed similar proliferation and migration ability as HUVECs. The SCAP-derived ECs could promptly generate new blood vessels and well anastomose with the host circulation in the presence of SCAP. The results of this study corroborated that the small molecule cocktail can efficiently reprogrammed SCAP into a functional endothelial lineage. This pioneering study on direct reprogramming dental stem cells into endothelial cells by chemical molecules may provide a promising protocol for producing sufficient ECs for vascular engineering and treatment of ischemic diseases. However, the large-scale expansion and maintenance of these induced ECs and the mechanism behind chemical induction remain to be addressed in our future work.

## Conclusion

The present study demonstrated that the combination of small molecules and growth factors could induce SCAP into endothelial-like cells. These cells exhibited some key endothelial characteristics and could generate blood vessels in vivo, which may provide a promising cell source for vascular engineering and treatment of ischemic diseases.

## Supplementary Information

**Additional file 1: **Appendix figures and tables and methods and materials**.**

**Additional file 2.**

**Additional file 3.**

## Data Availability

The datasets that support our conclusions of the current study are available from the corresponding author on reasonable request.

## Abbreviations

BMP-4: Bone morphogenetic protein-4
DPSCs: Dental pulp stem cells
ECs: Endothelial cells
EGM-2: Endothelial growth medium-2
HUVECs: Human umbilical vein endothelial cells
SCAP: Stem cells from apical papilla
SHED: Stem cells from human exfoliated deciduous teeth
SM: Small molecules
VEGF: Vascular endothelial growth factor
