# Supplementary material for: Conversion of stem cells from apical papilla into endothelial cells by small molecules and growth factors
Source: Stem Cell Res Ther. 2021 May 3;12:266. doi: 10.1186/s13287-021-02350-5 (PMC8091697; doi:10.1186/s13287-021-02350-5)
Supplement: Supplementary file 1 — Additional file 1: Appendix figures and tables and methods and materials. [file 13287_2021_2350_MOESM1_ESM.docx]

**Appendix Figures and Legends**

**
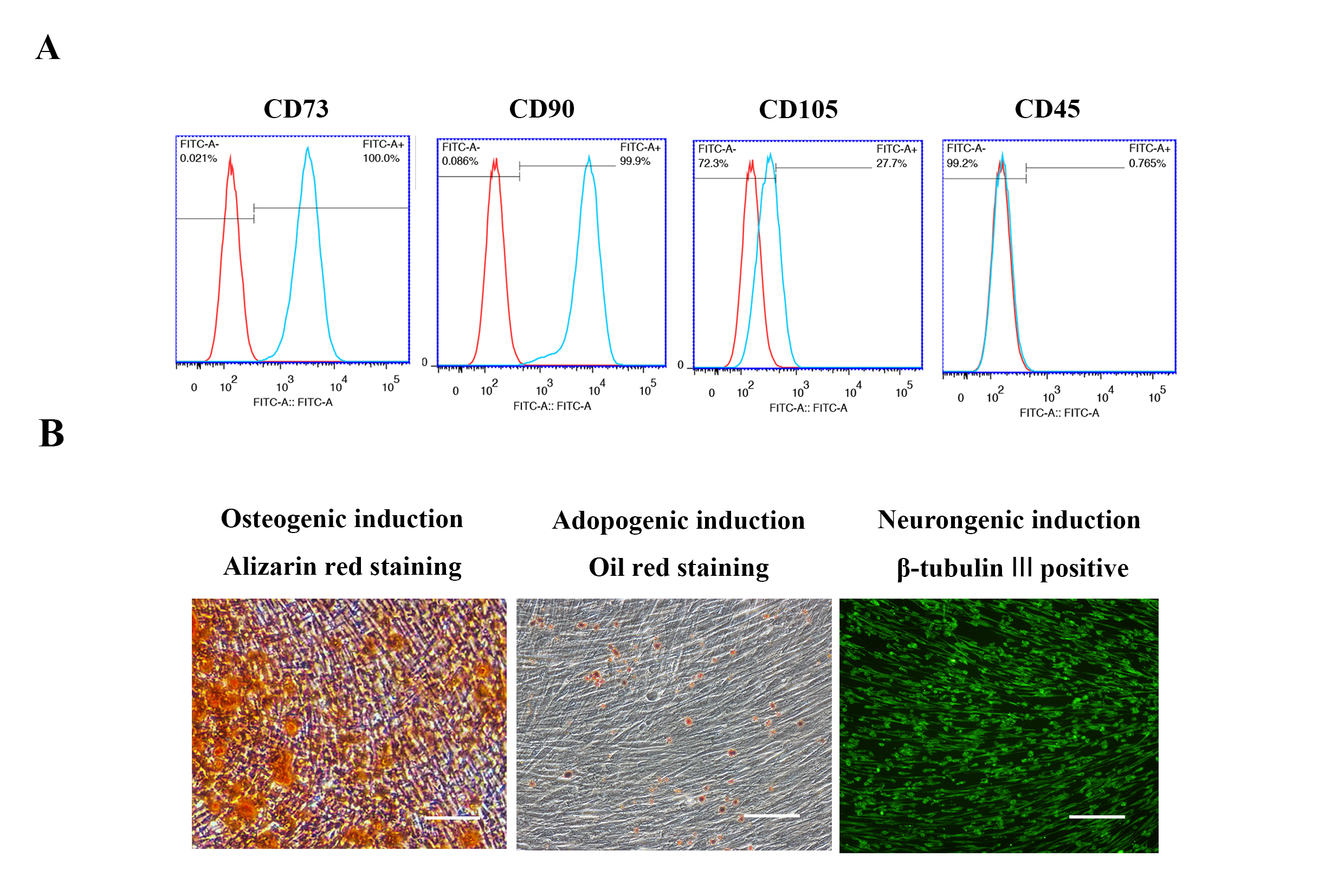
**

**Appendix Figure 1.** Characterization of SCAP. (A) Flow-cytometric analysis of cell surface antigens of SCAP. (B) Lineages differentiation of SCAP into adipo-, osteo-, and neural cells were tested. Scale bar = 200 μm.

**
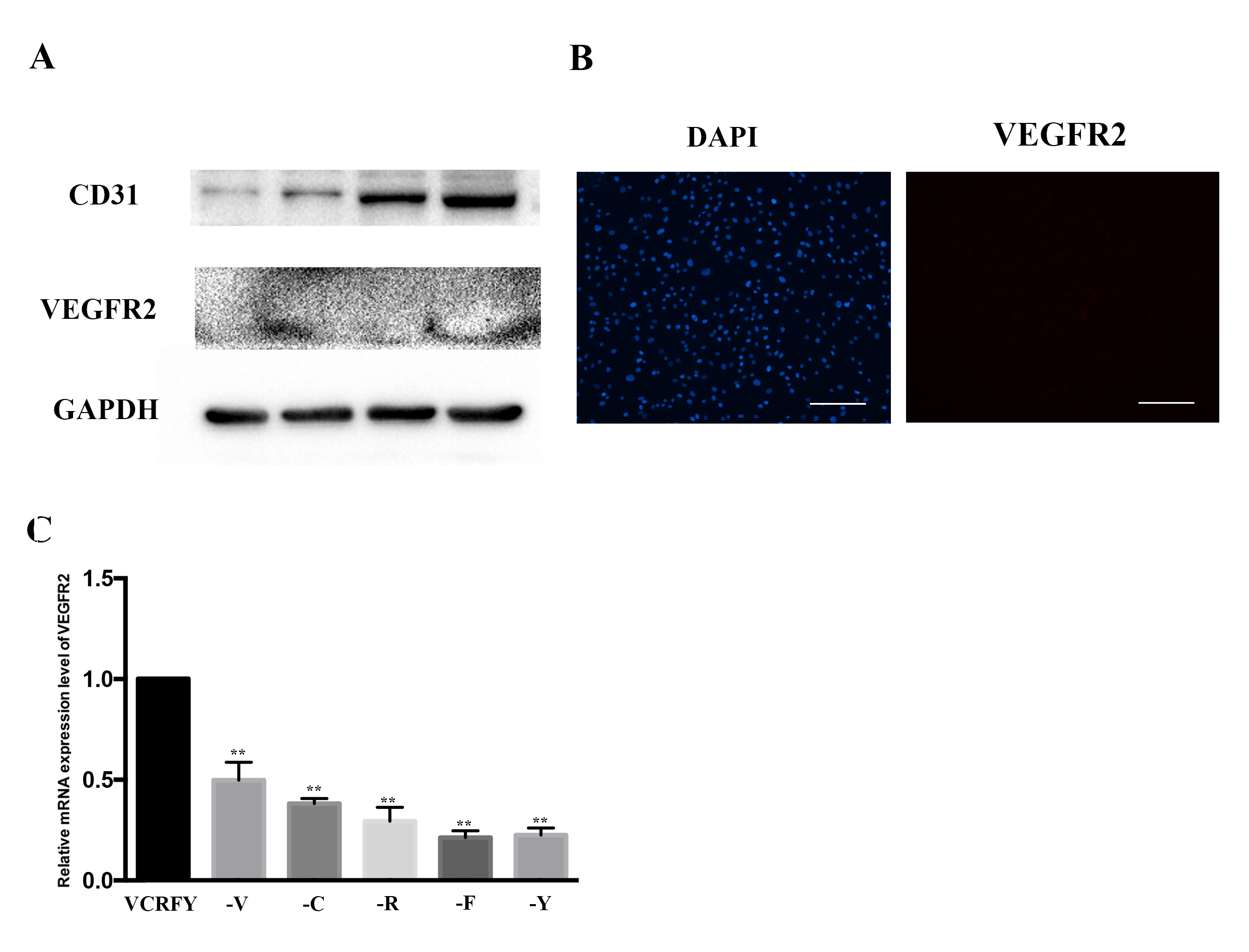
**

**Appendix Figure 2.** (A) The protein expression level of CD31, VEGFR2 of SCAP during SM exposure was assessed using Western blotting. GAPDH was used as an internal control. (B) The induce cells were immune-negative for VEGFR2. Scale bar =200 μm. (C) ) The gene expression levels of VEGFR2 after withdrawal of each small molecule (-VPA, -CHIR99021, - Repsox, - Forskolin, - Y-27632). All of the results represent the mean ± SEM of 3 independent experiments (n = 3). ^*^ *P* < .05 ^**^ *P* < .01.

**
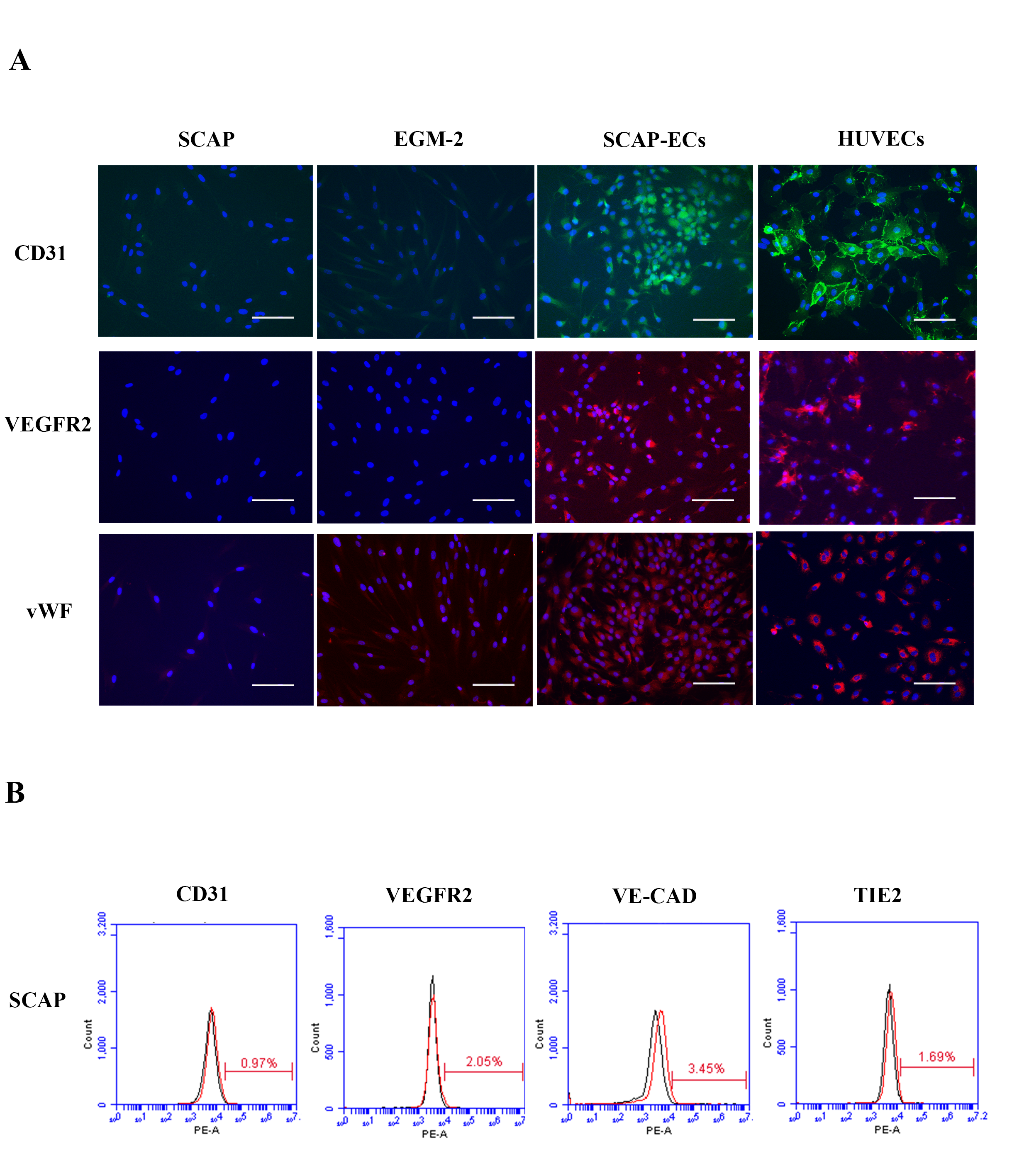
**

**Appendix Figure 3.** (A) Immunofluoresce analyse of endothelial markers in non-treated SCAP, SCAP in EGM-2, SCAP-ECs and HUVECs. The small molecule cocktail could significantly enhance the protein expression level of EC-specific proteins CD31, VEGFR2 and vWF compared with the non-treatment and EGM-2 groups. Scale bar = 100 μm (B) Flow-cytometric analyse of endothelial markers in non-treated SCAP. SCAP showed a relatively low expression of CD31, VEGFR2, VE-CADHERIN and TIE2.

**
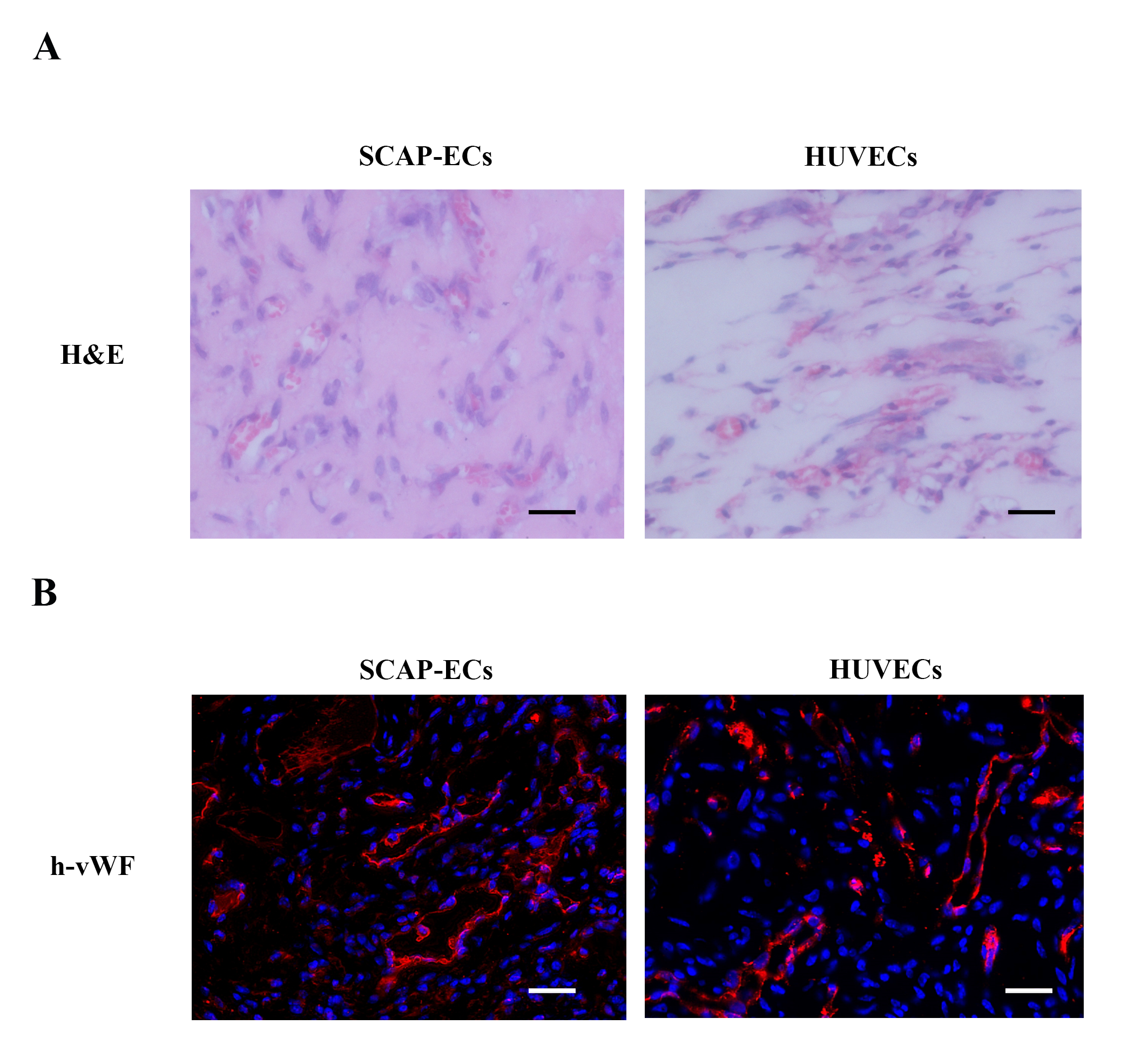
**

**Appendix Figure 4.** (A) Higher magnification images of H&E stained sections of Matrigel plugs of SCAP-ECs and HUVECs. (B) Higher magnification images of Immunofluorescence stained sections of Matrigel plugs SCAP-ECs and HUVECs for anti-human vWF (red). Nuclei were counterstained with DAPI (blue). All images are representative from 3 animals each group and 2 implants per mice. Scale bar = 25 μm

**Methods and Materials**

**1. Quantitative real-time polymerase chain reaction**

Total RNA was extracted using the RNeasy Plus Mini Kit (Qiagen, Valencia, CA, USA) following the manufacturer’s instructions. The concentration of RNA was quantified with Nanodrop 2000 spectrophotometer (NanoDrop Technologies, Wilmington, DE). Subsequently, 1 μg of total RNA was reverse transcribed for cDNA in a total reaction mixture of 20 µL system using Super-Script VILO Master Mix (Invitrogen, Carlsbad, CA). qRT-PCR analysis was performed on the Step-One Real-Time PCR System (Applied Biosystems, Grand Island, NY, USA) with SYBR Select Master Mix (Applied Biosystems, Grand Island, NY, USA). The primer sequences are shown in in Appendix Table 2. The measured mRNA levels were normalized against the endogenous GAPDH gene, and the 2-ΔΔCt method was used to compute the relative cycle threshold (Ct) values for each gene. Three experimental replicates were analysed for each gene.

**2. Western blotting**

To assess the expression of EC-specific proteins, western blotting was performed. The total cellular protein was harvested using RIPA protein lysis buffer (Thermo-Fisher Scientific, Carlsbad, CA, USA) containing protease inhibitor (Thermo-Fisher) and incubated on ice for 30 minutes. The concentration of extracted cellular proteins were quantified using the BCA kit (Pierce, Rockford, IL, USA). 30 µg of protein were electrophoresed in 8% SDS-PAGE gels (Bio-Rad, Hercules, CA, USA), and the blotted to polyvinylidene fluoride membrane (GE Healthcare Life Sciences, Little Chalfont, UK). The membranes were then blocked with 5% (w/v) skimmed milk at room temperature for 1 hour before incubation with primary and secondary antibodies, and the antibodies used are listed in in Appendix Table 3. The Pierce ECL chemiluminescent substrate (Thermo-Fisher) was used to visualize blots. The ImageJ 1.47v software (National Institutes of Health, Bethesda, MD, USA) was applied to quantify the band blots. Taken together, there were 3 experimental replicates for each protein analyzed.

**3. Flow cytometry analysis**

The SCAP-ECs were dissociated into a single-cell suspension. Subsequently, the cells were fixed and permeabilized using Cytofix/CytopermTM Fixation/Permeabilization Kit (BD Biosciences, San Jose, CA, USA). PE-conjugated primary antibodies were used to incubate cells on ice in the dark. The antibodies are listed in in Appendix Table 3. An isotype-matched antibody was used as a negative control. Flow cytometry data was analyzed with BD Accuri C6 software (BD Biosciences).

**4. Immunofluorescence analysis**

The SCAP-ECs cultures were washed 3 times with PBS, fixed with 4% paraformaldehyde and blocked with 5% bovine serum albumin at room temperature for 1 hour. The cover glass bottom dishes of induced endothelial cells were incubated with the following primary antibody at 4°C overnight: CD31 (Abcam, Cambridge, UK), VEGFR2 (Cell Signalling Technology, Danvers, MA), vWF (Sigma). After 3 washes with PBS, the cells were incubated with Alexafluor-594-conjugated secondary antibody (Invitrogen, Carlsbad, CA) and then stained with DAPI. The cover glass bottom dishes were mounted with FlourSave mounting reagent (Merck Millipore, Darmstadt, Germany) and the images were captured with an Olympus IX-81 confocal laser scanning microscope (Olympus, Tokyo, Japan).

**5. RNA sequencing analysis**

Total RNA was extracted using Trizol reagent (Invitrogen, CA, USA) following the manufacturer’s instructions. Quantity and purity of the extracted RNA were analysed using Bioanalyzer 2100 and RNA 1000 LabChip Kit (Agilent, CA, USA) with RNA number > 7.0. RNA samples were then converted into cDNA libraries with the use of Illumina (San Diego, CA). Subsequently, the paired-end sequencing on Illumina HiSeq 4000 instrument was performed following the vendor’s recommended protocol. EC-genes were selected with the criteria of an absolute expression level >3 FPKM in either HUVEC or SCAP-EC samples with at least 2-fold higher expression in SCAP-ECs or HUVECs than in SCAP only group. Gene list relative enrichments for various functional associations were determined using upregulated genes of SCAP-ECs versus SCAP. RNA sequencing data have been uploaded to the Gene Expression Omnibus database under the accession number GSE149930.

**6.Hematoxylin and Eosin (H&E) staining**

The sections were deparaffinized by two washes with xylene, then rehydrated with two 100% alcohol washes, two 95% alcohol washes, two 70% alcohol washes, and a brief wash with distilled water sequentially. The filtered Hematoxylin was used to cover the slides for 5 min. Then the sections were washed twice with distilled water (15s for each) to remove excess stains. After washing, the slides were immersed in Eosin for 1 min and repeated the washing step. After mounting, imaging was carried out under inverted microscope (Nikon Eclipse LV100N POL, Tokyo, Japan).

**7. Statistical analysis**

All experiments had at least technical replicates and were performed 3 times independently. All the results are presented as mean and standard deviation. Statistical analysis was carried out with Student’s t test for assays done between groups and 1-way ANOVA for multiple comparisons. *P* < 0.05 was considered statistically significant.

**Appendix Table 1.** Small molecule compounds and growth factors used for endothelial lineage conversion

| **Small**  **Molecule** | **Mechanism** | **Concentration** | **Source** | **Catalog#** |
| --- | --- | --- | --- | --- |
| Valproic acid (VPA) | HDAC inhibitor | 0.5mM | Sigma | P4543 |
| Repsox | TGFβ inhibitor | 1μM | Selleck | S7223 |
| CHIR99021 | GSK3 inhibitor | 3μM | Stemgent | 04-0004-10 |
| Forskolin | cAMP activator | 10μM | Caymen | 66575-29-9 |
| Y-27632 | ROCK inhibitor | 5μM | Sigma | Y0503 |
| rhVEGF_165_ | Promote the endothelial differentiation | 50ng/ml | Peprotech | 100-20 |
| BMP-4 | Mesoderm commitment | 20ng/ml | R&D | 314-BP-050 |
| 8-Br-3,5-cAMP | Promote the endothelial differentiation | 100μM | Sigma | B5386 |

**Appendix Table 2.** Primer sequences utilized in qRT-PCR

| Gene | Primer sequences | |
| --- | --- | --- |
| *CD31* | F | 5’-AAGCTGCCGGTTCTTAAATCC-3’ |
|  | R | 5’-AACTTGGTGGAAGGAGGGTATG-3’ |
| *VEGFR2* | F | 5’-CACAGTGGCCGACACCTAAA-3’ |
|  | R | 5’-TCTGACCATGTTGGCCAGACT-3’ |
| *VEGFR1* | F | 5’-TCCACCAAGCTCTAAATCCAAAC-3’ |
|  | R | 5’-CTGTCACAGGTGGTTTGCGTAT-3’ |
| *TIE2* | F | 5’-AGAAGCGGCCTAGGACAGAAC-3’ |
|  | R | 5’-CCTTTGGCGGAGGCATGTTT-3’ |
| *GAPDH* | F | 5’-TGCACCACCAACTGCTTAGC-3’ |
|  | R | 5'-GGCATGGACTGTGGTCATGAG-3’ |
| *IL-6* | F | 5’-TGCAATAACCACCCCTGACC-3’ |
|  | R | 5’-AGCTGCGCAGAATGAGATGA-3’ |
| *IL-8* | F | 5’-GGTGCAGTTTTG CCAAGGAG-3’ |
|  | R | 5’-TTCCTTGGGGTCCAGACAGA-3’ |

**Appendix Table 3.** Antibodies used for Western Blot, immunofluorescence and FACS analysis.

| Antibody | Source | Catalog # | Purpose | Host Species & Reactivity | Concentration |
| --- | --- | --- | --- | --- | --- |
| CD31 | Abcam | ab32457 | WB | Rabbit anti-human | WB: 1:1000 |
| CD31 | R&D | BBA7 | IF | Mouse anti-human | IF: 1:200 |
| CD31 | Abcam | ab256569 | IF | Rat anti-mouse | IF: 1:200 |
| VEGFR-2 | Cell Signalling Technology | 2479 | WB, IF | Rabbit anti-human | WB: 1:1000  IF: 1:200 |
| VE-cadherin (CD144) | Abcam | ab33168 | WB | Rabbit anti-human | WB: 1:1000  IF: 1:200 |
| vWF | Sigma | F3520 | IF | Rabbit anti-human | 1:50 |
| CD31-PE | R&D | FAB3567P | FC | Mouse anti-human | 10µL/10^6^ cells |
| VEGFR2-PE | R&D | FAB357P | FC | Mouse anti-human | 10µL/10^6^ cells |
| CD144 -PE | R&D | FAB9381P | FC | Mouse anti-human | 10µL/10^6^ cells |
| TIE2-PE | R&D | FAB3131P | FC | Mouse anti-human | 10µL/10^6^ cells |
| IgG-PE | R&D | IC002P | FC | Mouse IgG1, κ Isotype Control | 10µL/10^6^ cells |
| IgG-PE | R&D | IC0041P | FC | Mouse IgG2B  Isotype Control | 10µL/10^6^ cells |
| VCAM-1 | Abcam | ab134047 | WB | Rabbit anti-human | WB: 1:1000 |
| ICAM-1 | Abcam | ab53013 | WB | Rabbit anti-human | WB: 1:1000 |
