# Supplementary material for: Conversion of stem cells from apical papilla into endothelial cells by small molecules and growth factors
Source: Stem Cell Res Ther. 2021 May 3;12:266. doi: 10.1186/s13287-021-02350-5 (PMC8091697; doi:10.1186/s13287-021-02350-5)
Supplement: Supplementary file 2 — Additional file 2. [file 13287_2021_2350_MOESM2_ESM.html]

xml version="1.0" encoding="utf-8"?CEFirst Log 

|  |
| --- |
| CEFirst Log |
| Version : 8.3.4 |
| USER | BGPC000028812P |

---

# Hyphenated Words

Please check the following hyphenated words for consistency:

- endothelial-induction [spaced 1, hyphen 1]
- RNA-sequencing [spaced 1, hyphen 1]
- Sigma-Aldrich [hyphen 6, spaced 1]
- ac-LDL [hyphen 8]
- anti-human [hyphen 1]
- Bennaceur-Griscelli [hyphen 1]
- beta-catenin [hyphen 1]
- b-FGF [hyphen 1]
- blood-derived [hyphen 1]
- Bousse-Kerdiles [hyphen 1]
- Boyer-Di [hyphen 1]
- Br-cAMP [hyphen 6]
- Canul-Chan [hyphen 1]
- capillary-like [hyphen 1]
- cardiomyocyte-like [hyphen 2]
- cell-cell [hyphen 1]
- cell-derived [hyphen 2]
- cell-like [hyphen 1]
- chemical-induced [hyphen 1]
- cocktail-based [hyphen 2]
- Coleal-Bergum [hyphen 1]
- concentration-dependent [hyphen 1]
- Cortez-Toledo [hyphen 1]
- De-la [hyphen 1]
- dental-derived [hyphen 2]
- EC-like [hyphen 1]
- EC-related [hyphen 1]
- EC-specific [hyphen 4]
- endothelial-like [hyphen 5]
- endothelial-specific [hyphen 6]
- E-stained [hyphen 1]
- ex-vivo [hyphen 1]
- gelatin-coated [hyphen 1]
- human-specific [hyphen 1]
- immune-deficient [hyphen 1]
- immune-positive [hyphen 1]
- large-scale [hyphen 2]
- long-term [hyphen 1]
- low-density [hyphen 3]
- mesenchymal-to [hyphen 3]
- mouse-specific [hyphen 1]
- multi-lineage [hyphen 1]
- Nic-Can [hyphen 1]
- non-induced [hyphen 1]
- Non-pluripotent [hyphen 1]
- non-treated [hyphen 4]
- non-treatment [hyphen 4]
- pre-chilled [hyphen 1]
- pro-adhesive [hyphen 1]
- pro-inflammatory [hyphen 3]
- real-time [hyphen 1]
- re-plating [hyphen 2]
- Rho-associated [hyphen 1]
- Rodas-Junco [hyphen 1]
- Rojas-Herrera [hyphen 1]
- RT-PCR [hyphen 3]
- SCAP-derived [hyphen 8]
- SCAP-EC [hyphen 1]
- SCAP-ECs [hyphen 64]
- self-assembling [hyphen 1]
- serum-free [hyphen 1]
- single-cell [hyphen 1]
- SM-based [hyphen 1]
- TGF-beta [hyphen 1]
- TGF-β [hyphen 3]
- time-dependent [hyphen 1]
- tissue-specific [hyphen 1]
- toll-like [hyphen 1]
- tube-like [hyphen 6]
- tubular-like [hyphen 1]
- Twenty-four [hyphen 1]
- VE-CADHERIN [hyphen 2]
- VEGF-induced [hyphen 1]
- vWF-positive [hyphen 1]
- week-old [hyphen 1]
- Yla-Herttuala [hyphen 1]
- α-MEM [hyphen 2]
- β-catenin [hyphen 1]

---

Copyright © 2013-2021 SPi Global, Chennai, India
